# Mechanisms of Drug Resistance in Veterinary Oncology—A Review with an Emphasis on Canine Lymphoma

**DOI:** 10.3390/vetsci2030150

**Published:** 2015-08-12

**Authors:** Maurice Zandvliet, Erik Teske

**Affiliations:** Department of Clinical Sciences of Companion Animals, Faculty of Veterinary Medicine, Utrecht University, Yalelaan 108, 3508 TD, Utrecht, The Netherlands; E-Mail: E.Teske@uu.nl

**Keywords:** cancer, dog, multi-drug resistance, chemotherapy, P-gp, MRP1, BCRP, non-Hodgkin, therapy

## Abstract

Drug resistance (DR) is *the* major limiting factor in the successful treatment of systemic neoplasia with cytotoxic chemotherapy. DR can be either intrinsic or acquired, and although the development and clinical implications are different, the underlying mechanisms are likely to be similar. Most causes for DR are pharmacodynamic in nature, result from adaptations within the tumor cell and include reduced drug uptake, increased drug efflux, changes in drug metabolism or drug target, increased capacity to repair drug-induced DNA damage or increased resistance to apoptosis. The role of active drug efflux transporters, and those of the ABC-transporter family in particular, have been studied extensively in human oncology and to a lesser extent in veterinary medicine. Methods reported to assess ABC-transporter status include detection of the actual protein (Western blot, immunohistochemistry), mRNA or ABC-transporter function. The three major ABC-transporters associated with DR in human oncology are ABCB1 or P-gp, ABCC1 or MRP1, and ABCG2 or BCRP, and have been demonstrated in canine cell lines, healthy dogs and dogs with cancer. Although this supports a causative role for these ABC-transporters in DR cytotoxic agents in the dog, the relative contribution to the clinical phenotype of DR in canine cancer remains an area of debate and requires further prospective studies.

## 1. Introduction. Treatment Failure *versus* Drug Resistance

Chemotherapy is one of the major therapeutic modalities in human and veterinary oncology and the treatment of choice for systemic malignancies including hematopoietic tumors and metastatic cancers. In most cases cytotoxic drug treatment is initially successful, however long-term disease control is uncommon due to a failing responsiveness of the tumor to the treatment. 

In oncology treatment failure can be defined as failure to obtain a complete disappearance of tumor mass and/or associated paraneoplastic syndromes or recurrence following an initial complete response to a specific treatment. Although failure to respond to treatment is often considered synonymous with drug resistance (DR), there are other causes for treatment failure.

A well-known cause for treatment failure, and as a result apparent DR, is the difference between the expected effect (efficacy) of a therapy and the observed effect (effectiveness). This discrepancy partly results from the fact that efficacy is based on the results obtained from studies performed under ideal circumstances e.g., laboratory tests or highly controlled clinic trials, while effectiveness is established in clinical practice. Here the effects of genetic variation, patient co-morbidities, environmental effects, treatment decisions made by the clinician and patient-owner come into play. The net result is that effectiveness is typically lower than expected efficacy, but this does not represent DR. Other causes for apparent treatment failure include inappropriate choice of drugs, drug dosages or treatment intervals and also these iatrogenic causes should not be classified as DR.

Treatment failure commonly results from DR of the tumor cells, and this represents a major problem in medical oncology and limits the long-term successful use of all medical therapies. In most cases, DR will develop over time and under treatment (acquired DR), but it may already be present from the start of treatment (intrinsic DR). Although both situations represent two clinically distinct entities, the mechanisms underlying both of these situations are likely to be similar. Furthermore, it should be realized that the distinction between intrinsic and acquired DR is to some extent artificial and affected by the method used to establish a complete treatment response. Methods vary in sensitivity and range from establishing the absence of clinical signs based on patient history, physical exam and/or diagnostic imaging (clinical response), to disappearance of tumor cells in biological samples (cytologic response), or demonstrating the disappearance of neoplastic DNA or specific tumor-associated DNA mutations using PCR-based techniques (cytogenetic or molecular response). Treatment response in veterinary oncology is typically based on clinical response, and this does not necessarily imply a complete cytologic or cytogenetic response. If the vast majority of tumor cells is responsive to treatment, this will lead to the observation of a complete clinical response, but if a small subpopulation of intrinsic DR tumor cells persists, these cells will over time give rise to the recurrent tumor cell population and in this way lead to the false impression of acquired DR. 

Although DR is not limited to the classical cytotoxic drugs and also applies to the targeted cancer drugs, like protein-kinase inhibitors, resistance mechanisms to these drug classes are beyond the scope of this review.

## 2. Drug Resistance-Mechanisms

The DR phenotype can develop through a variety of mechanisms that can be roughly subdivided into two main categories: failure to reach sufficiently high drug levels at the tumor site or, despite sufficiently high drug concentrations at the tumor site, failure to achieve the appropriate (cellular) response.

Failure to reach therapeutic drug levels in the tumor can have iatrogenic, host- and tumor-related causes. Iatrogenic causes include inappropriate drug dose or treatment intervals and do not represent true DR, but only create an impression of DR. Host-related factors leading to DR include poor drug absorption, changes in systemic drug metabolism (either reduced drug activation or increased drug inactivation), increased drug clearance, and insufficient drug delivery to the tumor due to the presence of specific organ-barriers (e.g., the blood-brain barrier). Well-known tumor-related causes are insufficient perfusion of the tumor itself, the tumor microenvironment (e.g., hypoxia, acidosis, changes in redox-potential) [1], and the degree of non-proliferating (quiescent) tumor cells [2].

**Figure 1:**
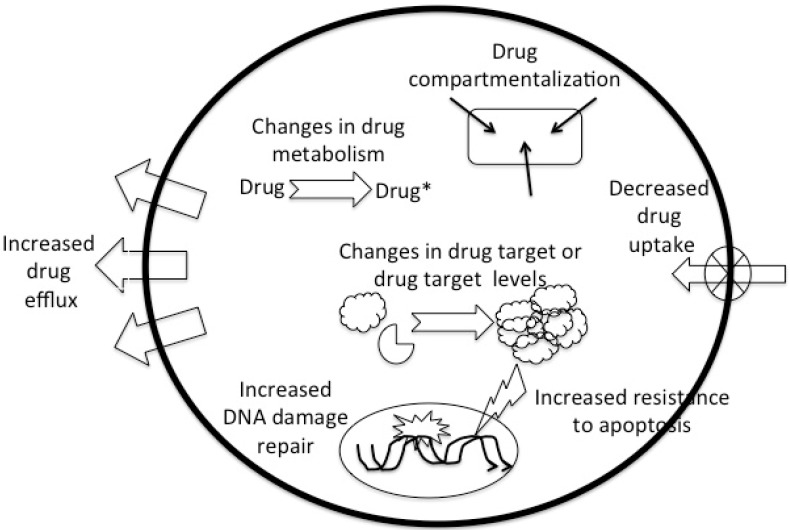
The major cellular drug resistance mechanisms.

Although the above-mentioned pharmacokinetic causes for DR occur and are clinically relevant, DR more often has a pharmacodynamic cause and most commonly finds its origin in the tumor cell. Well-known causes include decreased cellular drug uptake or increased drug excretion or drug compartmentalization, and changes in cellular drug metabolism (reduced activation or increased inactivation). Failure to achieve sufficient cellular response can result from increased repair of drug-induced DNA damage, increased resistance to apoptosis and changes in the drug target or changes (quantitative or qualitative) in drug target (Figure 1). 

The mechanisms underlying tumor cell DR can be specific for a given drug or drug class, but for most drugs multiple resistance mechanisms have been identified (Table 1) that can be simultaneously present. In most cases DR is not restricted to a single drug or drug class, but involves resistance to multiple structurally and chemically unrelated drugs, and is often referred to as multidrug resistance (MDR). MDR can result from the induction of a single resistance mechanism that is capable of handling multiple drugs, but it is also possible that a single drug triggers the development of multiple resistance mechanisms enabling the cell to handle multiple drugs.

The information available on DR in the dog is still limited, but in contrast to pharmacokinetic causes for DR that may vary between animals due to species-specific differences in drug metabolism, cellular mechanisms are likely to be similar to those in humans due to the conserved nature of the pathways involved.

**Table 1:** Major drug resistance mechanisms identified for the classical cytotoxic agent drug classes.

Mechanism	Drugs
Decreased uptake	methotrexate, other antimetabolites, nitrogen mustard, melphalan, cisplatin
Changes in drug metabolism (changes in activation or inactivation)	many antimetabolites (e.g., 5-fluorouracil, cytosine arabinoside), alkylating agents, cisplatin
Increased efflux (including compartmentalization)	methotrexate, melphalan, vinca-alkaloids, taxanes, etoposide, anthracyclines
Modifications in target enzyme	methotrexate, other antimetabolites, topoisomerase inhibitors
Increased DNA repair	alkylating agents, cisplatin, anthracyclines, etoposide
Resistance to apoptosis	alkylating agents, cisplatin, anthracyclines, etoposide

## 3. Development of Drug Resistance

DR is a stable or permanent characteristic for a given tumor and is generally assumed to have a genetic basis. The assumption of a genetic background is based on the observation that drug resistant clones generate spontaneously at a rate consistent with known rates of genetic mutation and that this rate can be further increased by exposing tumor cells to mutagenic compounds. Furthermore drug resistant cells retain their resistance in the absence of the initiating drug. The fact that drug resistant clones in a tumor arise through spontaneous mutations forms the basis for the Goldie-Coldman hypothesis [3] that states that the probability of having at least one drug resistant cell within a tumor cell population depends on tumor size. The relation between tumor size and probability of cure (*i.e.*, the absence of resistance) can be described by: P = e^−αN^ (P: probability of cure, α: spontaneous mutation rate per cell division, N: number of tumor cells). Assuming a mutation rate per cell division of 1 × 10^−6^, the chance of having a DR clone within 1 mm^3^ tumor mass (±10^6^ cells) is > 60%.

This model predicts that in order to maximize treatment results, chemotherapy should be initiated when the tumor is at its smallest size (small N), meaning either treat the disease early or in an adjuvant setting (e.g., chemotherapy following surgical removal of the tumor). This model also predicts that a multidrug protocol that uses a combination of drugs with different and independent resistance mechanisms has a higher efficacy than a monodrug protocol. Assuming that the likelihood of resistance for a tumor cell to drug A is 1/a and that to drug B 1/b, the chance of a tumor cell being resistant to both these drugs is 1/(a × b).

There are two major theories as to how DR could arise and while one suggests selection as the driving force, the other assumes the presence of an innate drug resistant subpopulation within each tumor. The selection theory assumes that DR results from the repeated exposure of tumor cells to cytotoxic drugs in a process similar to that of evolution. As Howard Skipper demonstrated in mouse models [4], cytotoxic drugs are incapable of inducing a 100% cell kill and the intermittent exposure to cytotoxic drugs combined with the genetic variation and genetic instability within a tumor, offers a selective advantage to those tumor cells that are less susceptible to the cytotoxic effects of the administered drugs. This continuous selection will ultimately lead to the emergence of a “new” resistant tumor cell population and acquired DR.

The second theory postulates a subpopulation of intrinsic DR cancer cells within each tumor and these cells are now referred to as cancer initiating cells or cancer stem cells. Cancer stem cells are pluripotent DR cancer cells that retain the essential property of self-protection through, amongst others, increased activity of multidrug transporters [5]. These quiescent constitutively DR cancer stem cells remain present in low frequency within the heterogeneous tumor and serve as a reservoir for DR tumor relapses. 

Although DR is thought to have a genetic basis resulting from gene mutation and/or amplification, it has more recently been shown that epigenetic regulation (DNA hypermethylation, histone deacetylation, microRNAs) plays an important role as well [6,7].

## 4. Pharmacodynamic Mechanisms Associated with Drug Resistance

### 4.1. Decreased Drug Uptake

The uptake of drugs into (tumor) cells occurs through passive diffusion (e.g., doxorubicin, vinblastine), facilitated diffusion and active transport (e.g., nucleoside analogs). Cytotoxic drugs can enter a cell along a concentration gradient in all three ways, but only active transport allows for uptake against a concentration gradient. Most plasma membrane transporters belong to the Solute Carrier family (SLC) [8] and decreased drug uptake can result from either a reduced binding affinity of a drug to its transporter or a reduced number of transporters. Both mechanisms have been described, the former for melphalan (L-type amino acid transporters/SLC7A5 [9]), the latter for methotrexate (reduced folate carriers/SLC19A1 [10]) and the nucleoside analogues cytarabine, fludarabine and gemcitabine (reduced nucleoside transporters/SLC28 and SLC29 [11]). 

In the dog there are no data available on the role of reduced drug uptake in relation to DR.

### 4.2. Changes in Drug Metabolism

Drug metabolizing enzymes are important determinants of both systemic and intracellular drug concentrations. Although oxidation, reduction and hydrolysis (Phase I reactions) and conjugation (Phase II reactions) play a crucial role in protecting normal cells against toxins, these same reactions can also lead to DR in cancer cells due to either decreased activation of prodrugs (decreased enzyme activity, reduced affinity to activating enzyme) or increased inactivation of drugs (increased enzyme activity) [12].

The most important Phase I enzyme is the cytochrome P450 (CYP) system and DR due to increased drug inactivation was reported for docetaxol in human breast cancer where a low CYP3A4 expression led to a better treatment response. Reduced drug activation is a cause for DR for many of the alkylating agents including cyclophosphamide that has to be activated in the liver.

Phase II reactions include conjugation of drugs to glucuronic acid, sulfate and glutathione (GSH), which improves drug excretion, reduces drug activity and detoxifies reactive electrophilic drugs. An example of a Phase II enzyme is the glucuronidation enzyme uridine 5′-diphosphate-glucuronosyl transferase that is associated with inactivation of anthracyclines and topoisomerase I inhibitors. Glutathione transferases (GSTs) conjugate GSH to toxic electrophilic compounds. These include both endogenous metabolites resulting from oxidative stress as well as exogenous xenobiotics, like cytotoxic agents. Increased GSH- and GST-mediated detoxification plays a role in the resistance to many alkylating agents, platinum drugs and doxorubicin [13]. For some drugs both reduced activation and increased inactivation have been described and for instance for 5-fluoruracil (5-FU) both reduced expression and/or activity of the activating enzyme thymidine synthase and increased activity of the inactivating enzyme dihydropyrimidine dehydrogenase (DPD) were all associated with a lower sensitivity to 5-FU.

Other examples in altered drug metabolism include reduced intracellular methotrexate retention due to decreased polyglutamation (either due to decreased folylpolyglutamate synthase activity or increased γ-glutamatehydrolase activity [14]), cytarabine resistance due to decreased kinase or increased deaminase activity [11], and irinotecan resistance due to loss of activating carboxy-esterase-2 activity.

The heavy metal scavenger metallothionein can bind and inactivate certain platinum complexes and reactive-oxygen species and elevated expression, for instance in response to hypoxia, is associated with a poor response to platinum agents [15]. Metallothionein has been detected in canine primary lung carcinoma [16]. 

### 4.3. Increased Drug Efflux

Drug efflux is often referred to as the Phase III system and given the wide range of substrates these transporters can handle, increased efflux capacity is potentially the most important DR mechanism. The first efflux pump discovered was P-glycoprotein (P-gp) with the P standing for permeability. P-gp, also known as multidrug resistance protein 1 (MDR1) or ABCB1, proved to be the first member of the ATP-Binding Cassette (ABC) superfamily, a group of proteins that uses ATP-hydrolysis to actively transport substances across biological membranes. Other important members of the ABC-superfamily associated with DR include the multidrug resistance-associated proteins 1 (MRP1; ABCC1) and 2 (MRP2; ABCC2) and breast cancer resistance protein (BCRP; ABCG2), which will be discussed in more detail in Section 5. 

Although ABC-transporters are typically located in the outer cell membrane, allowing for transport of substrates out of the cell, they can also be found in organelle membranes (endoplasmic reticulum, mitochondria and peroxisome), which enables compartmentalization of drugs and other xenobiotics, a process known as sequestration. Cytotoxic drugs can be transported from the cytosol and sequestered in the endoplasmatic reticulum where they no longer exert their cytotoxic effect leading to DR [17]. The expression of TAP (transporter associated with antigen processing; ABCB2) has been associated with mild resistance to doxorubicin, vincristine and etoposide [18].

Although most transporter proteins belong to the ABC superfamily, there are ATP-dependent non-ABC transporters associated with DR including lung-resistance protein (LRP) and RLIP76, also known as RALBP1. LRP was identified as the major vault protein (MVP), the main component of multimeric vault particles, and is thought to confer DR by regulating nucleocytoplasmic transport and cytoplasmic redistribution of drugs away from their cellular targets [19]. Although LRP has been detected in canine cancers [16,20], there are no data on the relative contribution of LRP to DR in the dog. RLIP76 is capable of transporting a wide variety of cytotoxic drugs, including doxorubicin and its glutathione-conjugates [21] in humans, but there are currently no data on the role of this transporter in the dog.

### 4.4. Changes in Drug Target

Changes in drug target are an important mechanism for DR to antimetabolites, vinca-alkaloids and topoisomerase inhibitors. These drugs interact with intracellular protein targets and resistance is either due to an increase in target protein or a mutation in the target protein resulting in a reduced drug affinity without loss of its normal biologic activity. Examples include resistance to methotrexate (increased dihydrodrofolate reductase (DFHR) expression and mutated DFHR with a reduced affinity), and 5-FU (increased thymidylate synthase expression [22]). Microtubules are assembled from α-tubulin and β-tubulin heterodimers and overexpression or mutation of β-tubulin isotypes is associated with DR to tubulin-binding agents like paclitaxel [23]. Topoisomerases catalyze topological changes in the DNA structure, necessary for DNA duplication and RNA transcription, by causing a temporary single strand (topoisomerase I) or double-stranded (topoisomerase II) break in the DNA. Topoisomerase inhibitors inhibit either of these two enzymes, by forming stabilized DNA-topoisomerase complexes, preventing re-ligation of the nucleotide strands and inducing apoptosis. Resistance to topoisomerase I inhibitors (campothecin, topotecan) and topoisomerase II inhibitors (doxorubicin, mitoxantrone, etoposide), results from reduced topoisomerase expression or the development of a mutated topoisomerase with no or reduced drug affinity [24].

Although it seems likely that similar DR mechanisms occur in the dog, this has not yet been reported.

### 4.5. Repair of Drug-Induced DNA Damage

Cells are constantly subjected to potential causes for DNA damage, including oxidative stress, chemicals and radiation, leading to ±20,000 DNA lesions per day. Cells have developed defense mechanism against reactive oxygen species and are capable of repairing DNA damage [25]. While loss of DNA repair mechanisms increase the risk of developing mutations and ultimately cancer, an increased repair capacity reduces cells’ susceptibility to the growth inhibitory effects of cytotoxic drugs. DNA repair mechanisms include direct base repair (O^6^-methyl-guanine DNA methyltransferase; MGMT), nucleotide excision repair (NER), base excision repair (BER; abasic sites, single-strand break repair), base pair mismatch repair (MMR) and homologous recombination repair (HRR) and non-homologous end-joining (NHEJ) for DNA double-strand break repair and interstrand crosslink repair. Increased DNA repair has been linked with DR to alklyating agents, platinum drugs and topoisomerase inhibitors.

Increased expression of the NER enzyme ERCC1, is commonly identified in cisplatin-resistant cancer cells [26]. Alkylating agents cause a covalent modification (methylation or chlorethylation) at the O^6^-position of guanine (chlorambucil, lomustine and temozolamide) or the N^7^-position of purine bases (cyclophosphamide, iphosphamide) [27]. The major repair-mechanism for monofunctional alkylation of the O^6^-guanine base is through the base repair enzyme O^6^-methyl-guanine DNA methyltransferase (O^6^-MGMT), that removes the alkyl group from the O^6^-guanine position and transfers it to a specific cysteine residue on the enzyme, inactivating itself in the process [28]. O^6^-MGMT levels and methylation status of the gene’s promotor region (epigenetic regulation) are useful predictors for the response to alkylating agents in gliomas [29] and B-cell lymphoma [30]. Bifunctional alkylating agents use MGMT for removing the primary adduct followed by NER (including ERCC1) and homologous recombination. Increased DNA-dependent protein kinase (essential for nonhomologous end-joining repair) and XRCC3 (homologous repair) expression have been associated with resistance to melphalan [31]. 

DNA repair mechanisms have been studied in canine mammary tumors, mast cell tumors and B-cell lymphomas. Up-regulation of the DNA-repair enzymes *BRCA2* and RAD51 has been demonstrated in canine mammary carcinomas [32,33]. MMR enzyme expression (MLH1, MSH2, MSH6) has been detected in canine mast cell tumors, but proved independent of tumor grade and was not related to treatment response [34]. No relation was found between O^6^-MGMT mRNA levels (RT-qPCR) and treatment response to chemotherapy (corresponding to intrinsic DR) in canine B-cell lymphoma [20].

### 4.6. Resistance to Apoptosis

Apoptosis results from activation of the extrinsic pathway (death receptor-mediated; Fas/CD95) or the intrinsic pathway (mitochondrial-mediated). Although chemotherapy-induced activation of the extrinsic pathway has been described [35], chemotherapy more often activates the intrinsic pathway resulting in the release of mitochondrial cytochrome c that together with APAF-1 and caspase-9 leads to the formation of the apoptosome. The intrinsic pathway is complex and regulated by many factors including proteins of the Bcl-2 family, activating and inhibiting proteases, reactive oxygen species, Ca^2+^ and ceramide [36,37].

Reduced sensitivity and DR could theoretically result from mutations in the targets upstream of the mitochondria (including p53, Akt and RAS), the mitochondrial level (most importantly members of the Bcl-2 family), and those downstream of the mitochondria, including heat shock proteins, the inhibitor of apoptosis survivin, caspase-3 deletion and caspase-independent factors, like apoptosis inducing factor (AIF) [37]. 

DNA-damage and other forms of cellular stress lead to stabilization of p53, cell cycle arrest and either DNA repair or apoptosis. Loss-of-function mutations in the p53 gene are amongst the most commonly identified mutations in a wide variety of cancers in both humans and animals. Cell lines with p53 mutations, including lymphoma cell lines, have a poorer response to treatment with cytotoxic agents resulting in a DR phenotype [38]. The reported immunohistochemical expression of p53 in canine lymphoma varies from low [39] to common [40] and was found to be higher in T-cell lymphomas compared to B-cell lymphomas. Although inactivating p53 mutations have been demonstrated in primary and relapsed canine lymphomas, these could not be linked to DR [20,41,42].

The balance between pro- and anti-apoptotic members of the Bcl-2 family controls mitochondrial membrane integrity and overexpression of the anti-apoptotic Bcl-2/Bcl-x_L_ proteins correlates with chemoresistance in human leukemia [43], breast cancer [44] and chondrosarcoma [45]. Bcl-2 expression (mRNA) failed to relate to treatment response in canine B-cell lymphoma [20].

Survivin inhibits apoptosis and increased survivin expression was associated with a shorter disease-free period in dogs with multicentric B-cell lymphoma, but failed to increase following relapse [46].

Although modulating apoptosis appears a promising route in cancer treatment [37], there are conflicting results regarding the importance of resistance to apoptosis in clinical DR [47,48].

DR cannot only result from resistance to apoptosis, but also from up-regulation of pro-survival pathways of which the Ras/Raf/MEK/ERK, PI3K/PTEN/Akt/mTOR and NF-kB signaling pathways are important representatives. Dysregulation of these signaling pathways often results from mutations in upstream growth factor receptors and/or associated protein kinases, which could potentially be inhibited with targeted agents and small molecule inhibitors [49].

## 5. ATP-Binding Cassette Transporters

### 5.1. History

The ABC-transporter superfamily is one of the largest and most well conserved protein families in biology and its members are found in almost all life forms. Despite their importance, the first ABC-transporter was not discovered until 1976 when the expression of Permeability glycoprotein or P-gp (ABCB1) was first described in a multidrug resistant Chinese hamster ovary cell line selected for colchicine resistance [50]. 

Initially P-gp was considered to be the main cause for DR in general, including cytotoxic drugs, but in later years other ABC-transporters were identified in DR cancer cell lines [51,52]. It took almost 20 years before, by accident, the physiological function of P-gp was identified. The death of a colony of *mdr1a (-/-)* knock-out mice following treatment with ivermectin for a skin mite infection led to the discovery that the ivermectin neurotoxicity resulted from the absence of P-gp in the blood-brain barrier [53]. A few years later history repeated itself, when the high susceptibility of the Scottish collie breed to ivermectin-induced neurotoxicosis could be related to a deletion mutation in the *mdr1* gene [54].

### 5.2. ABC-Transporters

So far 48 ABC-transporters have been described in humans that are classified into seven subfamilies labeled A through G [55] (http://nutrigene.4t.com/humanabc.htm). Characterization of the ABC-transporter proteins is based on the sequence and organization of their ATP-binding domain(s) or nucleotide-binding folds (NBF) that contain the characteristic Walker A and B motifs. A functional ABC-transporter consists of at least two NBFs located in the cytoplasm that hydrolyze ATP and transfer the energy, and two trans-membrane domains that enable substrate transport across the membrane.

ABC-transporters are capable of exporting a wide variety of substrates, both of exogenous origin (xenobiotics) including drugs and toxins, as well as endogenous origin including phospholipids, ions, peptides, steroids, polysaccharides, amino acids, organic anions and bile acids. Because of the essential role of ABC-transporters in many physiological processes, it is not surprising that loss-of-function mutations in ABC-transporters can give rise to a wide variety of genetic diseases including cystic fibrosis (chloride ions; ABCC7), adrenoleukodystrophy (very long chain fatty acids; ABCD1), progressive familial intrahepatic cholestasis (phospholipids and bile acids; ABCB4 and ABCB11), Dubin-Johnson syndrome (conjugated bilirubin; ABCC2), Stargardt disease (N-retinylidene-phosphatidyl-ethanolamine; ABCA4), Tangier disease (cholesterol; ABCA1), immune deficiencies (ABCB2, ABCB3), *Pseudoxanthoma elasticum* (ABCC6), and persistent hyperinsulinemic hypoglycemia of infancy (ABCC8, ABCC9) [56].

### 5.3. Assessing ABC-Transporter Expression and Function

The role of ABC-transporters can be assessed at three levels, *i.e.*, the expression of the specific protein (immunohistochemistry, Western blot), its mRNA (RT-qPCR,) and the function (dye efflux studies). In order to demonstrate that DR results from an increase in drug transport capacity, quantifying ABC-transporter function would be considered most appropriate. However, this requires the availability of a fresh, single-cell tumor cell suspension. These requirements make this assay practically challenging to perform with patient samples, but nevertheless its use has been described in hematologic tumors. 

Immunohistochemistry on tumor biopsies is considered a good alternative to function testing, but there is not necessarily a fixed relation between transporter protein expression and transporter function or capacity. A second problem with immunohistochemistry is that most commercially available antibodies are directed against the human or murine ABC-transporters and might not cross-react with those of other species or are not specific enough to detect the various ABC-transporter variants.

Assessing mRNA expression is technically less demanding than immunohistochemistry, requires small tumor samples, is semi-quantitative and primers can be relatively easy generated for the different ABC-transporters and animal species.

It has been shown that ABC-transporter mRNA levels in cell lines do not always predict protein expression [57] or *in vitro* drug sensitivity [58]. Furthermore ABC-transporter expression or function in human tumor samples does not necessarily predict *in vivo* chemosensitivity [59,60]. Information on these relations in veterinary oncology is limited, but the relation between P-gp function and chemosensitivty has been reported in canine cell lines [61,62].

### 5.4. ABC-Transporters in Pharmacology

High expression levels of the various ABC-transporters are found in the intestine, liver, and kidney where they determine drug uptake and elimination, as well as in barrier tissues, like the blood-brain barrier placenta and stem cells where they affect drug distribution [63] (Table 2).

Another way by which ABC-transporters affect a drug’s pharmacological profile is through so-called drug-drug interactions, which result from the concomitant use of multiple drugs that are substrates for the same ABC-transporter. Substrate competition can lead to reduced elimination resulting in increased, and potentially toxic, drug levels. Although this might seem farfetched, multi-drug therapies or simultaneous treatments for multiple diseases are common in both humans and animals. Furthermore it should be realized that drugs used for routine preventative medical care [64] or herbal medicines [65] can be ABC-transporter substrates and have similar effects.

**Table 2:** Overview of distribution of the ABC transporters P-gp, MRP1, MRP2 and BCRP in selected human barrier tissues [55,63].

Tissue	P-gp	MRP1	MRP2	BCRP
Lung	apical	basolateral	not detected	apical
Intestine				
Duodenum	apical	basolateral	apical	apical
Jejunum	apical	basolateral	apical	apical
Ileum	apical	basolateral	apical	apical
Colon	apical	basolateral	apical	apical
Liver	apical	basolateral	not detected	apical
Kidney	apical	basolateral	apical	not detected
Brain				
BBB	apical	apical	apical	apical
BCSFB	apical	basolateral	-	-
Testis	apical	basolateral	not detected	not detected
Placenta	apical	basolateral	apical	apical

BBB = blood-brain barrier, BCSFB = blood-cerebrospinal fluid barrier.

Over the last few years it has become clear that ABC-transporter polymorphisms can account for the variation in drug response observed within a population. Single-nucleotide polymorphisms (SNPs) have been demonstrated for P-gp, MRP1, and BCRP, of which some can result in changes in transporter expression or function and affect drug absorption, excretion and distribution in humans [66]. This is likely to occur in animals as well, but so far no relevant SNPs have been established other than a deletion mutation for P-gp in some canine dog breeds [54].

### 5.5. ABC-Transporters in Drug Resistance

Although twelve of the 48 described ABC-transporters function as drug-efflux pumps, DR is typically associated with three ABC-transporters: P-gp (MDR1/ABCB1), multidrug resistance-associated protein 1 (MRP1/ABCC1) and breast cancer resistance protein (BCRP/ABCG2) [67].

**Figure 2:**
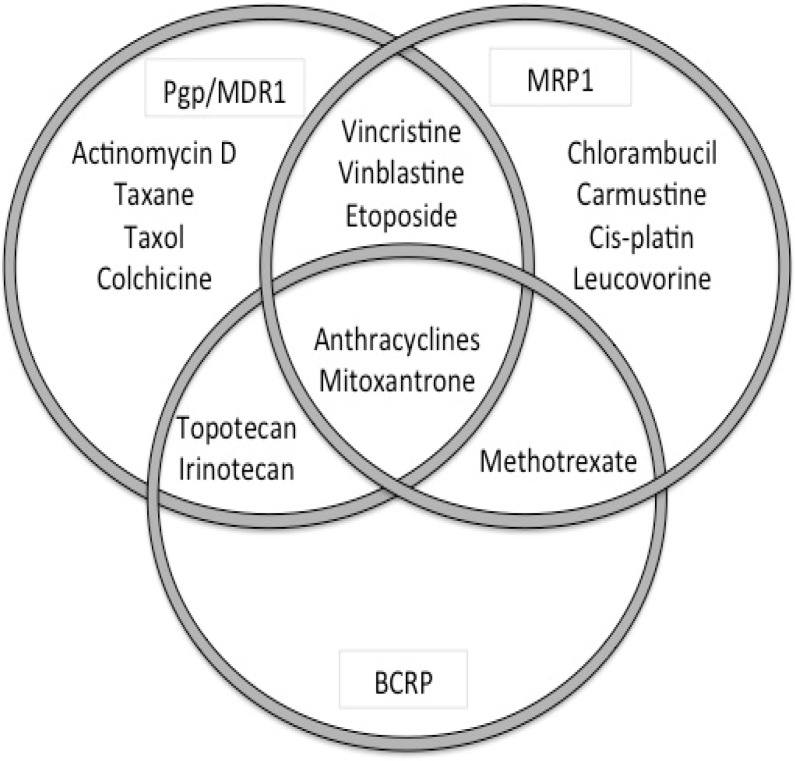
Overlap in cytotoxic dugs as substrates for the major ABC-transporters ABCB1 (P-gp), ABCC1 (MRP1) and ABCG2 (BCRP).

Interpreting data on the role of these transporters in DR can be challenging and results vary based on the techniques used (mRNA, protein, function), samples (cell line, tumor-sample), method of DR induction (exposure to substrate or transfection), and species differences. *In vitro* studies demonstrated that most ABC-transporters transport a wide range of substrates, but with a considerable degree of substrate overlap between the various transporters [68] (Figure 2, Table 3). Furthermore cancer cells can express several ABC-transporters simultaneously, although typically the expression of a single transporter is dominant [69].

Data on the role of ABC-transporters in DR to cytotoxic drugs in the dog are limited and much of what we know is based on *in vitro* studies in canine cell lines, but there are some data on ABC-transporter expression in canine neoplasia. 

**Table 3:** Overview of the relevant ABC transporters associated with drug resistance to cytotoxic agents in humans (adapted from Dean M. The Human ATP-Binding Cassette (ABC) Transporter Superfamily [Internet]. Bethesda (MD): National Center for Biotechnology Information (US); 2002 Nov 18).

Gene	Substrates	Inhibitors
ABCB1 (MDR1, P-gp)	colchicine, doxorubicin, vincristine, vinblastine, paclitaxel, etoposide, digoxin, saquinivir	verapamil, PSC833, GF120918 (GG918), V-104, Pluronic L61, LY335979, XR9576, OC144-093
ABCC1 (MRP1)	doxorubicin, daunorubicin, vincristine, vinblastine, etoposide, colchicine	Cyclosporin A, V-104, MK571
ABCC2 (MRP2, cMOAT)	vinblastine, sulfinpyrazone	PSC833, MK571
ABCC3	methotrexate, etoposide	
ABCC4	nucleoside monophosphates (thiopurines)	MK571
ABCC5	nucleoside monophosphates	
ABCG2 (BCRP)	mitoxantrone, topotecan, doxorubicin*, daunorubicin*, CPT-11	fumitremorgin C, Ko143, GF120918

* mutant BCRP

#### 5.5.1. ABCB1 or MDR1/P-gp

P-gp (ABCB1) or multi-drug resistance protein 1 (MDR1) is a 170 kDa transmembrane protein and was the first ABC-transporter to be identified [50]. 

In humans, P-gp is expressed at high levels in the apical membrane of epithelial cells including the small intestine, colon, liver and bile ducts, pancreatic ductules, kidney (proximal tubule), endothelial cells in the brain (luminal side), testes, inner ear, adrenal cortex, endometrium (during pregnancy), placenta and hematopoietic stem cells [70,71]. In humans, high pretreatment P-gp expression has been observed in hematopoietic malignancies (leukemia, lymphoma, multiple myeloma) and a variety of carcinomas including renal, colon, hepatocellular, adrenal, mammary and ovarian carcinoma [70]. 

P-gp is capable of transporting a wide variety of structurally unrelated substrates (Table 4), but they are typically neutral or cationic (at physiologic pH), lipid-soluble, organic compounds, often with aromatic rings, and a molecular weight of 200–1.900 Da. Endogenous substrates include steroid hormones (aldosterone, β-estradiol-17β-D-glucuronide), lipids (phospholipids, glycosphingolipids), peptides (β-amyloid peptides) and possibly small cytokines (IL-2, IL-4 and IFN-γ) [72]. Exogenous substrates include a wide variety of drugs including cytotoxic agents like the natural product-derived vinca-alkaloids, taxanes and anthracyclines.

P-gp polymorphisms have been described in a number of cancer types as well as their effect on (cytotoxic) drug therapy, but up to now no consistent pattern has emerged linking P-gp polymorphisms to DR [73].

**Table 4:** A selection of clinically relevant drugs and compounds that are known P-gp substrates [55,68].

Drugs Used in Oncology			Non-Oncological Drugs	
**Cytotoxic agents**	Anthracyclines	doxorubicin, daunorubicin, epirubicin	**Antibiotics**	doxycycline, erythromycin, rifampin, tetracycline
	Anthracenes	mitoxantrone, bisantrene	**Antifungals**	itraconazole, ketoconazole
	Antitumor antibiotics	actinomycin-D, mitomycin-C, plicamycin mithramycin)	**Antiparasiticides**	ivermectin, moxidectin, selamectin
	Taxanes	paclitaxel, docetaxel	**Antiemetics**	domperideone, ondansetron
	Topoisomerase I inhibitors (campothecins)	irinotecan, topotecan	**Antidiarrheal agents**	loperamide
	Topoisomerase II inhibitors (epipodophyllotoxins)	etoposide, teniposide	**Anticonvulsant drugs**	phenobarbital, phenytoin, levetiracetam
	Vinca-alkaloids	vincristine, vinblastine, vinorelbine, vindesine	**Cardiac drugs**	digoxin, diltiazem, quinidine, verapamil losartan
			**Antihistamines (H2-antagonsits)**	cimetidine, ranitidine, terfenadine
**Steroids**	Glucocorticoids	cortisol, dexamethasone, hydrocortisone, methylprednisolone, prednisolone, triamcinolone	**Immunosupppressants**	cyclosporine A, tacrolimus
	Mineralocorticoids	aldosterone	**Opioids**	butorphanol, morphine
	Sex steroids	estradiol, progesterone	**Miscellaneous**	amitryptiline, colchicine, phenothiazines
**Tyrosine kinase inhibitors**	imatinib, gefitinib			

P-gp is highly conserved between species and canine P-gp shows ±90% homology with human P-gp [74]. In the healthy dog the distribution of P-gp has been characterized using immunohistochemistry and RT-qPCR and expression was found to be high in the liver (canalicular side of hepatocyte), bile ducts, kidney (mostly epithelium of proximal tubule), pancreatic ducts, adrenal cortex and brain (endothelial cells) [75,76]. Lower levels were detected in the stomach, small intestine and colon (both apical surface and diffuse cytoplasmatic staining), lung (alveolar and bronchiolar epithelium) and lymphocytes (both B- and T-lymphocytes; membranous staining) [77]. P-gp was not detected in normal or hyperplastic mammary tissue [78,79]. A deletion mutation in the *MDR1* gene was found to lead to ivermectin-induced neurotoxicosis in the collie [54] but was later also described in other dog breeds [80] (Table 5) as well as other drug toxicoses, albeit all well-established P-gp substrates [81,82,83,84,85].

**Table 5:** Dog breeds with a high frequency of multidrug resistance (MDR)-1 gene deletion [80].

Collie	Shetland sheepdog
Old English sheepdog	Australian shepherd
White German shepherd dog	Miniature Australian shepherd
English shepherd	Silken windhound
Longhaired whippet	McNab

P-gp expression has been described in canine cancer cell lines, including lymphoid leukemia, mast cell tumor and osteosarcoma cell lines [61,86,87], as well as clinical tumor samples, including hepatic, adrenal, gastrointestinal, mammary pulmonary and transitional cell carcinoma, mast cell tumors and malignant lymphoma [16,75,78,79,88,89,90,91].

In canine cell lines P-gp expression has been associated with DR to doxorubicin and vincristine [62,74,86], although mRNA levels in tumor samples failed to correlate with *in vivo* doxorubicin sensitivity [74].

#### 5.5.2. ABCC1 or MRP1

Multidrug resistance protein 1 (MRP1) or ABCC1 is a 190 kDa membrane-bound protein and was identified in 1992 in a multidrug resistant lung cancer cell line [51]. MRP1 is typically found on the basolateral membrane of polarized epithelial cells in the intestinal mucosa, kidney (limb of Henle, collecting ducts), brain (choroid plexus), testes, and bone marrow [63]. MRP1 has been found to be overexpressed *in vitro* in a number of drug-selected cell lines including leukemia, lung, breast, bladder, prostate, and cervical cancer cell lines, as well as *in vivo* in human patients with leukemia and a variety of solid tumors, including gastrointestinal tract, (non-small cell) lung, breast, ovarian and prostate carcinomas and melanoma [92].

MRP1 is a transporter for both hydrophobic and water-soluble uncharged compounds (Table 6) including glutathione, glutathione-conjugates (leukotrienes, prostaglandins), glucuronide conjugates (β-estradiol-17β-D-glucuronide, glucuronosyl-bilirubin), sulfate conjugates (dehydroepiandrosterone-3-sulfate, sulfatolithocholyl-taurine) and heavy metal oxyanions including arsenite and trivalent antimonite [68]. MRP1 has been associated with resistance to natural anticancer drugs including vincristine (but not taxanes), doxorubicin, epirubicin, etoposide and their conjugated metabolites, methotrexate, and the GSH-conjugated metabolites of alkylating agents. 

**Table 6:** A selection of clinically relevant drugs and compounds that are known Multidrug Resistance Protein 1 (MRP1)-substrates [55,68].

Drugs Used in Oncology			Non-Oncological Drugs	
**Cytotoxic agents**	Anthracyclines	doxorubicin, daunorubicin, epirubicin, idarubicin	**Antibiotics**	difloxacin grepafloxicin
	Topoisomerase I inhibitors (campothecins)	irinotecan, topotecan	**Metalloids**	arsenite trivalent antimonite
	Topoisomerase II inhibitors (epipodophyllotoxins)	etoposide, teniposide	**Peptides**	glutathione
	Vinca-alkaloids	vincristine, vinblastine, vinorelbine, vindesine	**Glutathione conjugates**	cyclophosphamide-SG, doxorubicin-SG melphalan-SG,
	Antifolates	methotrexate	**Sulfate conjugates**	dehydroepiandrosterone-3-sulfate estrone-3-sulfate,
**Tyrosine kinase inhibitors**	imatinib, gefitinib		**Glucuronide conjugates**	estradiol-17-β-D-glucuronide, etoposide-glucuronide, irinotecan-glucuronide,
			**Folates**	folic acid, L-leucovorin

Although over 50 MRP1 polymorphisms have been described, only a few of these are thought to potentially affect drug response [93].

MRP1 shows a high degree of homology between species, but tissue expression can vary significantly between species. For instance MRP1 is present in dog and rat, but not or only limited in human and monkey hepatocytes [94]. In the dog a high MRP1 expression is found in the brain, kidney, liver and testes, while lower levels are found in the lungs, intestines [76] and lymphocytes [77].

In dogs with cancer MRP1 expression has been reported in multicentric B-cell lymphoma [20], cutaneous mast cell tumors [89] and solid tumors including pulmonary [16], hepatocellular [95], transitional cell [90] and mammary carcinomas [96]. Canine MRP1 is associated with resistance to vincristine and etoposide, but not anthracyclines [97].

#### 5.5.3. ABCG2 or BCRP

ABCG2, also known as Breast Cancer Resistance Protein (BCRP) or mitoxantrone resistance protein (MXR), is a 72 kDa membrane-bound protein and was first described in 1998 [52]. ABCG2 is expressed in epithelial cells (apical side) of the small intestine, colon, kidney (renal tubular cells), liver (canalicular side of hepatocyte), brain (luminal side of capillary endothelial cells), retina, mammary glands (during pregnancy and lactation), placenta [98] and pluripotent hematopoietic stem cells [99].

BCRP can transport both positively and negatively charged drugs, as well as their respective sulfate conjugates (Table 7), and reported substrates include mitoxantrone, methotrexate [100], campothecin, topotecan, etoposide and tyrosine kinase inhibitors [101]. BCRP does not transport vinca-alkaloids, taxanes, doxorubicin (only mutant BCRP [102]) or cisplatin.

**Table 7:** A selection of clinically relevant drugs and compounds that are known Breast Cancer Resistance Protein (BCRP)-substrates [55,68].

Drugs Used in Oncology			Non-Oncological Drugs	
**Cytotoxic agents**	Anthracyclines	doxorubicin (mutant form), daunorubicin	**Antibiotics**	ciprofloxacin, norfloxacin, nitrofurantoin
	Anthracenedione	mitoxantrone, bisantrene (mutant form)	**Anthelmintics**	albendazole, oxfendazole
	Topoisomerase I inhibitors (campothecins)	irinotecan, topotecan	**Diuretics**	furosemide, hydrochlorothiazide
	Topoisomerase II inhibitors (epipodophyllotoxins)	etoposide, teniposide	**Porphyrins**	pheophorbide A, protoporphyrin IX, hematoporphyrin
	Antifolates	methotrexate	**Flavonoids**	genestein, quercetin
**Tyrosine kinase inhibitors**	imatinib, gefitinib, lapatinib		**Fungal toxins**	alfatoxin B, fumitremorgin C, Ko143
			**Drug & metabolite compounds**	acetaminophen-sulfate, estrone-3-sulfate, dehydroepiandrosterone-3-sulfate, estradiol-17-β-D-glucuronide, dinitrophenyl-S-glutathione

BCRP expression has been described in hematopoietic tumors (leukemia, lymphoma [103]) and solid tumors of the gastrointestinal tract, endometrium, lung and melanoma [104]. BCRP expression appeared to be more common in T-cell lymphomas [105].

A number of BCRP polymorphisms have been described of which some lead to reduced expression and/or function, while others result in changes in substrate specificity [102,106].

Species differences in BCRP expression have been described and BCRP expression has for instance been detected in human and dog hepatocytes, but not in those of mouse and monkey [94]. BCRP expression was demonstrated in canine mammary cancer cell lines [107], canine lymphoma (higher in T-cell than B-cell lymphoma [108]) and benign and malignant canine mammary tumors (higher expression in malignant tumors and expression increases with tumor grade [96,109]).

A study with cloned canine cDNA demonstrated that canine BCRP mediates resistance to doxorubicin [96], which is different from the situation in humans where doxorubicin transport was only described for a mutant form of human BCRP [102].

### 5.6. Induction and Regulation of ABC-Transporter Expression

The induction of ABC-transporters, and P-gp in particular, has been studied extensively and can result from a variety of environmental stimuli such as exposure to xenobiotics (including carcinogens and cytotoxic drugs), hypoxia, heat shock, irradiation and inflammation [110].

The pregnane X receptor (PXR) and constitutive androstane receptor (CAR) are both members of the nuclear receptor superfamily that also includes the thyroid, glucocorticoid and estrogen receptors. PXR and CAR function as sensors for xenobiotics and regulate their metabolism and clearance [111]. PXR (NR1I2), first identified in 1998 [112], is primarily activated by pregnanes, but also recognizes a wide variety of drugs including dexamethasone, rifampicin, spironolactone [113], tamoxifen and a variety of cytotoxic drugs including vinca alkaloids (vincristine, vinblastine), taxanes (paclitaxel, docetaxel) and alkylating agents (cyclophosphamide, ifosfamide) [114]. Activation of PXR and CAR leads to the up-regulation of multiple target genes including Phase I (CYP3A4, CYP2B6) and Phase II enzymes (UDP-glucuronosyltransferases, sulfotransferases), as well as ABC-transporters. PXR was shown to induce expression of P-gp, MRP3 and OATP1A2, while CAR was associated with P-gp, MRP2, MRP3 and MRP4 (ABBC2, ABCC3, ABCC4) expression [115]. Increased PXR expression has been associated with resistance to paclitaxel and cisplatin in HEC-1 cells [116] and doxorubicin in human colon adenocarcinoma cells [114]. PXR inhibitors would offer a promising new therapeutic option, unfortunately designing a specific and non-toxic inhibitor appears challenging [116].

Many ABC-transporter substrates are capable of inducing ABC-transporter expression, for instance through PXR or CAR, and in the case of the substrate being a drug, administration of the drug can have profound effects on its own pharmacokinetic and pharmacodynamic behavior as well as its effects, which could in the case of cytotoxic drugs lead to the induction of DR. This theory has been suggested as the most likely cause for the poorer treatment results obtained with chemotherapy in dogs with multicentric lymphoma that have been pretreated with glucocorticoids prior to starting chemotherapy [117,118,119,120]. Although P-gp expression in the dog was shown to increase following exposure to endogenous substances, e.g., increased bile acids [121], drugs, including prednisolone [122] and rifampicin [123], and following a spontaneous status epilepticus [124], it has not yet been demonstrated for cytotoxic drugs or in neoplastic cells.

The regulation of ABC-transporter expression has also been linked to several classical oncogenes and tumor suppressor genes including p53, the Ras/Raf pathway, the APC (adenomatous polyposisi coli) gene, and possibly c-Jun NH2-terminal kinase (JNK). Although wild-type p53 is a repressor of the P-gp and MRP1 genes, several mutant p53 proteins are capable of activating the P-gp promotor [125]. Furthermore, P-gp is also one of the target genes of the Ras/Raf signaling pathway [126] and inactivating APC mutations activate the P-gp promotor [127]. The role of the MAPK/ERK and JNK pathways in the regulation of ABC-transporter expression has been studied in canine lymphoid cell lines. And while activation of the MAPK/ERK pathway resulted in up-regulation of the P-gp expression, activation of JNK led to a decrease in P-gp expression [128,129]. Activation of the MAPK/ERK and JNK pathway both resulted in a decreased BCRP expression [130].

ABC-transporter expression does not only result from genetic changes (gene rearrangements, mutations), but also epigenetic regulation and microRNA appear to be of importance [110]. In most canine lymphoma samples the CpG-island promotor of the P-gp gene was found to be hypomethylated with no significant difference in methylation status between drug sensitive and DR lymphoma samples [131]. As yet there is no information available on the role of microRNAs in canine lymphoma.

## 6. Drug Resistance due to ABC-Transporters in Canine Lymphoma

Although it is generally accepted that failure to respond to chemotherapy is the major cause for treatment failure in canine lymphoma, relatively little is known about the underlying mechanisms. In line with the situation in human oncology, most studies have focused on the role of ABC-transporters, and P-gp in particular, both *in vitro* in canine cell lines and *ex vivo* in tumor biopsy samples.

Although multiple canine lymphoid cell lines have been reported including a T-cell leukemia cell line (CL-1) [132] and B-cell lymphoma cell line (CLBL-1) [133], most studies have been performed in the canine B-cell leukemia cell line GL-1 [134]. In two separate studies DR to doxorubicin was established through exposure of GL-1 cells to increasing concentrations of doxorubicin [62,86]. Both studies demonstrated that DR was associated with increased P-gp expression, described cross-resistance with the P-gp substrate vincristine and reversal of DR by blocking P-gp function with P-gp blocking agents like verapamil or PSC833. A recent *in vitro* study with GL-1 cells described DR in relation to P-gp mRNA expression (RT-qPCR), P-gp protein expression (immunocytochemistry) and P-gp function (dye efflux studies) with all three techniques leading to similar results [62]. This study also evaluated the ABC-transporters MRP1 and BCRP, but these were found to be of little or no importance in this specific model. 

The older studies on ABC-transporter expression in clinical lymphoma samples focused on P-gp expression using immunohistochemistry. These studies found that in pretreatment samples P-gp expression was typically lower (3–33%) than at relapse [88,135,136]. Although some studies reported a prognostic effect of P-gp expression on DFP and survival [88,135], these findings could not be confirmed in a more recent study [137].

Using RT-qPCR to detect P-gp mRNA, it was found that P-gp mRNA expression in canine multicentric lymphoma [108] was typically low, while being higher in gastrointestinal lymphomas [138]. This is not surprising given the fact that gastrointestinal lymphomas are well-known for their poor response to chemotherapy and are likely to be intrinsically DR [139]. 

In a recent *ex vivo* study ABC-transporter expression (besides *ABCB1, ABCC1* and *ABCG2,* also the non-classical ABC-transporters *ABCB5, ABCB8, ABCC3,* and *ABCC5*) was measured at different time points throughout disease progression (pre-treatment, first relapse and clinically drug-resistant) and related to immunophenotype (B-cell *versus* T-cell) and the type of DR (intrinsic *versus* acquired) [108]. It was found that there were significant differences in ABC-transporter expression between B- and T-cell lymphomas, with T-cell lymphomas showing a higher *ABCB5* and *ABCC5*, and lower *ABCC1* expression compared to B-cell lymphomas. Intrinsic DR appeared more common in T-cell lymphomas and could not be related to a specific ABC-transporter. Acquired DR could be associated with increased *ABCB1* expression in B-cell lymphomas, while in T-cell lymphomas an increase in *ABCG2* expression was found. Relapse samples were not associated with significant increases in ABC-transporter levels, but this was not unexpected given the fact that in most relapse cases a second complete response was obtained with further chemotherapeutic treatment, implying that most relapses were not associated with DR.

Although pre-treatment P-gp, MRP1 and BCRP mRNA expression were not predictive of treatment outcome in dogs with multicentric lymphoma [20,108], P-gp mRNA expression in the peripheral blood was found to correlate with the likelihood of chemotherapy-related toxicity [140].

Given the fact that most studies in both dogs and humans are not able to consistently relate ABC-transporter expression to treatment response and prognosis (disease-free period, progression-free survival), it seems likely that other DR mechanism must play a role as well.

## 7. Glucocorticoids and Drug Resistance

Glucocorticoids (GCs) are stress-induced steroid hormones that, in the blood, are predominantly bound to corticosteroid-binding globulin. Due to their lipohilic nature CBG-free GCs can easily cross membranes by passive diffusion and once in the cytoplasm they bind to GC-receptors. The activated receptor then translocates into the nucleus, where it binds to GC response elements in the promotor regions of GC-responsive genes [141]. Activation of these GC-responsive genes leads to the well-known effects on metabolism, homeostasis and immune function.

Most (lymphoma) chemotherapy protocols use the potent synthetic GCs prednisolone and dexamethasone, but their use is limited by their side effects on metabolism and homeostasis (Cushing’s syndrome) and the development of GC resistance. The reason for using GC in the treatment of lymphoid neoplasia is best explained by looking at the effects of GC on the immune system. These can be summarized as induction of lymphoid cell apoptosis (activation of the intrinsic pathway), cell cycle arrest and inhibition of inflammation (repression of pro-inflammatory transcription factors including NF-κB and AP1). Of these three mechanisms, the induction of apoptosis appears most important and GCs can affect this on three levels: at the genomic level (induction of pro-apoptotic Bcl-2 family member Bim and the GC-induced leucine zipper), in the cytoplasm (increase in cytosolic calcium, ceramide, reactive oxygen species levels and net potassium efflux) and the actual execution of apoptosis (activation of caspase 9) [142]. Activation of these pathways has been demonstrated in human acute lymphoblastic leukemia [143].

GC resistance predominantly results from resistance to apoptosis, and several mechanisms have been identified [144] including insufficient ligand, changes related to the GC receptor (mutations, insufficient expression), deficiencies in the GC receptor-associated proteins, mutations in the apoptotic pathway, and activation of pro-survival pathways RAS/RAF/MEK/ERK, PI3K/PTEN/Akt/mTOR and Jak/STAT [145]. Insufficient ligands can result from impaired systemic GC uptake, increased GC–binding proteins, reduced bioactivation (only with prednisone), overexpression of drug efflux transporters (most commonly P-gp, MRP and LRP) and increased GC inactivation. *In vitro* GCs (dexamethasone) have been shown to induce DR through up-regulation of P-gp expression in lymphoid cells [146], and GC resistance (dexamethasone) was associated with increased PI3K activity as well as resistance to vinblastine and doxorubicin [147]. These findings provide a logical explanation for the observation that dogs with multicentric lymphoma that have been treated with GCs prior to starting a treatment with chemotherapy have a poorer prognosis. In this subgroup of animals it is well possible that the GCs were capable of inducing P-gp expression.

Although GCs have been successfully used in the management of canine lymphoid neoplasia for many years [148], it is surprising to note that neither dexamethasone nor prednisolone had an antiproliferative effect in the canine lymphoid cell lines CL-1 and GL-1 [62,149]. Resistance to the antiproliferative effect of GCs might result from down-regulation of the number of GC-receptors possibly through down-regulation of the NF-κB pathway [149]. Furthermore, a recent study failed to confirm that prednisolone was a substrate for canine P-gp, or that short-term prednisolone incubation lead to significant increases in *ABCB1*, *ABCC1* and *ABCG2* mRNA expression in both a doxorubicin sensitive and its derived doxorubicin resistant lymphoid cell line despite the presence of a functional GC-receptor [62]. Lastly, ABC-transporter mRNA expression did not increase in tumor samples taken from dogs with lymphoma that relapsed while receiving treatment with a doxorubicin-based chemotherapy protocol that included GCs [108].

Removing GCs from a doxorubicin-based chemotherapy protocol for the treatment of canine lymphoma did not negatively affect treatment outcome in the primary treatment, nor did it have any effect on treatment results obtained with additional chemotherapy following relapse [150]. In summary, all these data suggest that it seems unlikely that prednisolone induces DR through up-regulation of ABC-transporters (including P-gp), but it is not inconceivable that GC resistance through other mechanisms than upregulation of ABC-transporters, e.g., resistance to apoptosis, could coincide with DR to cytotoxic agents in dogs with lymphoma.

## 8. Targeting Drug Resistance

### 8.1. Prevention of Drug Resistance-Use of Cytotoxic Agents

Most tumors tend to become drug resistant to medical therapies and prevention or reversal of DR would be likely to result in improved treatment results and prognosis for cancer patients treated with chemotherapy.

Initial attempts to prevent drug resistance were based on the Goldie-Coldman hypothesis and aimed at preventing drug resistance by using multidrug chemotherapy protocols [151] or high dose chemotherapy [152,153]. The rationale behind this approach was to increase the overall cell killing and thereby prevent the development of drug resistant clones. Analysis of relevant clinical studies suggests that although multidrug and high-dose protocols result in better tumor responses, neither of these approaches prevents the emergence of DR. Furthermore this approach appears only effective in drug sensitive tumor types, like lymphoma and leukemia.

### 8.2. Modulation of Drug Resistance-ABC-Transporters Modulators

An alternative approach has been to inhibit individual DR mechanisms and since active drug efflux through ABC-transporters appears to be the most important mechanism, work has mainly focused on developing P-gp and other ABC-transporter inhibitors (Table 8). However, it has to be realized that ABC-transporters are not only present in cancer cells, but also in the liver, kidney, intestine and many tissue-barriers. Modulation of ABC-transporters (either though induction or inhibition) can therefore significantly affect absorption, distribution and excretion of drugs and affecting their pharmacokinetic and pharmacodynamics behavior potentially leads to either subtherapeutic or toxic drug levels. 

Within 10 years following the discovery of P-gp, the first trials with P-gp inhibitors were started. The first generation P-gp inhibitors were mostly drugs that were already used for other purposes (calcium-channel blockers, immunosuppressive agents) and although the *in vitro* studies with these inhibitors were promising, they proved generally ineffective *in vivo* due to their low inhibitory potency and significant toxicity at the concentrations required for sufficient P-gp inhibition. This led to the development of second-generation P-gp inhibitors that were more potent, but also less selective and often inhibited multiple ABC-transporters and sometimes even the cytochrome P450 system (CYP3A4). The inhibitory effect of these first- and second-generation P-gp inhibitors was demonstrated for a number of cytotoxic agents by changes in the pharmacokinetics (increased area under the curve) and pharmacodynamics (increased number of adverse events/toxicity) [154]. The lack of transporter specificity of these second-generation P-gp inhibitors caused pharmacokinetic interactions and affected the metabolism and clearance of cytotoxic drugs leading to unacceptable chemotherapy-related toxicity. The initial response was to lower the dose of the cytotoxic agents, but since the inhibitory effects on metabolism and clearance proved unpredictable, some patients were still overdosed, while others were underdosed and as a result therapeutic benefit was not always apparent [155]. Third-generation P-gp inhibitors are both more potent and more specific and hold promise, but the results of large-scale clinical trials are not yet available. There are no specific MRP1-inhibitors, and although there are “specific” BCRP-inhibitors, none of these have been evaluated in clinical trials. The use of a combined P-gp/BCRP inhibitor appears most interesting, but there are no data available on the clinical benefits.

**Table 8:** Modulators of the ABC-transporters P-gp, MRP1, and BCRP [68].

Generation	P-gp	MRP1	BCRP
First	verapamil quinidine cyclosporine A		Ko143 Pantoprazole Gefitinib? Imatinib? Quercetin?
Second	PSC833 (valspodar) VX-710 (biricodar)	VX-710 (biricodar)	VX-710 (biricodar)
Third	GF120918 (elacridar) XR9576 (tariquidar) LY335979 (zosuquidar) ONT–093 (ontogen)		GF120918 (elacridar) XR9576 (tariquidar)

Since the dog is commonly used in preclinical drug testing, many potential P-gp inhibitors have been evaluated in the dog [156]. P-gp mediated drug resistance was successfully reversed with the classical P-gp inhibitors verapamil, cyclosporin A, and PSC833, in a variety of canine cell lines [61,62,86,87,157]. The cyclosporine analogue PSC833 (Valspodar^®^) proved effective in overcoming P-gp mediated doxorubicin resistance *in vitro* (human osteosarcoma cell line) and was subsequently used in dogs with osteosarcoma [157]. PSC833 blood concentrations sufficiently high to block P-gp were obtained without causing significant toxicity and the combined use of PSC833 and doxorubicin allowed for significant doxorubicin dose reductions, however no data were reported on the clinical benefit of this combined use. A more recent study described the combined use of PSC833 and doxorubicin as a first-line treatment for dogs with multicentric B-cell lymphoma [158]. Although this study did not aim to demonstrate a potential treatment benefit (increase in duration of remission or survival), the reported survival data make such a benefit unlikely. Despite the fact that PSC833 failed to improve the outcome in humans with recurrent or DR multiple myeloma [159], it has to be realized that both veterinary studies describe the use of PSC833 in dogs with non-DR tumors, and it is therefore too early to conclude that PSC833 has no use in the treatment of dogs with (ABC-transporter mediated) DR lymphoma. 

### 8.3. Alternative Drugs and Therapies

Various other approaches to reverse DR have been described and include the use of small peptides designed to correspond to the transmembrane domain of P-gp [160], monoclonal antibodies or active immunization against P-gp [161,162], down-regulation of Pgp-expression [163] and gene silencing [164]. 

Over the past few years the combined use of classic cytotoxic agents with tyrosine-kinase inhibitors (TKIs) has gained interest as a potential new approach for overcoming DR. Tyrosine kinases play crucial roles in many of the pathways involved in cancer development, including cell proliferation, apoptosis, angiogenesis and metastasis and TKIs have been developed to specifically target these pathways. Resistance to TKIs is common and typically associated with P-gp and BCRP expression [165]. However TKIs are not only substrates for these specific ABC-transporters, but also inhibitors and the combined use of TKIs with cytotoxic agents has been successfully used in reverting drug resistance *in vitro* in a variety of human [166,167,168] and canine cell lines [169,170]. Both canine cell line studies used the same veterinary licensed TKI, masitinib-mesylate, but had different objectives. The first canine cell line study [169] demonstrated that the combined use of masitinib and a cytostatic drug led to a reduction in IC_50_ for a number of cytotoxic drugs in a variety of canine cancer cell lines, and this observation was referred to as the drug-sensitizing effect of masitinib. The second study [170] focused on DR in a canine lymphoid cell line and found that although masitinib itself was cytotoxic, its true value lay in its ability to inhibit P-gp function at concentrations achievable in the dog, and in this way revert P-gp mediated resistance to doxorubicin.

Other drugs that might be considered include proton-pump inhibitors [171] (the use of lansoprazole has been reported in veterinary cancer patients including dogs with DR lymphoma [172]), the MDM2 (mouse double minute) inhibitor nutlin-3 [173], and the Akt inhibitor perifosin [129].

## 9. Summary, Concluding Remarks and Future Studies

Resistance to chemotherapy is a multifactorial problem and in order to fully understand its complexity, aspects of general cancer biology (activation of pro-survival pathways, resistance to apoptosis, genomic instability, the existence of cancer stem cells), the tumor and its microenvironment, general pharmacology (pharmacokinetics and pharmacodynamics) and chemotherapeutic agents with their inherent failure to obtain 100% cell killing, will all need to be taken into consideration. Although *in vitro* data support an important role for the ABC-transporters in DR, they are unlikely to be the sole cause for *in vivo* DR in both humans and dogs. However, assuming a major role for ABC-transporters in DR, it is understandable why most canine lymphoma rescue protocols are based on the combination of alkylating agents, L-asparaginase and GCs, since none of these drugs are typical ABC-transporter substrates. The fact that these responses are typically short-lived suggests that at this stage often multiple DR mechanisms (next to ABC-transporter overexpression) are simultaneously present. 

In order to maximize tumor control and improve our treatment results for dogs with lymphoma, we will have to increase our understanding of DR in general and canine lymphoma in particular. This means that we will need to shift the focus of our research to mechanisms outside the field of classical pharmacology. Without knowing how many and which mechanisms will be important, it seems very likely that the relative importance of these mechanisms may vary between tumor types, between individuals with the same tumor type, and possibly even within the same tumor in the same patient over time. In order for this information to be useful in daily clinical practice and allow for personalized/precision, medicine, a fast and thorough histological, genetic and molecular characterization of a patient’s tumor is of the utmost importance. Technical limitations for performing these types of analyses are vanishing at a rapid pace and the genetic characterization of a tumor is within the financial reach of a pet owner, as well as potential medications for targeting these pathways. Although the more immediate challenge for the clinician lies in how to interpret these data and use them for making therapeutic decisions, they appear promising for the successful management of future patients.
